# Silencing of the ER and Integrative Stress Responses in the Liver of Mice with Error-Prone Translation

**DOI:** 10.3390/cells10112856

**Published:** 2021-10-23

**Authors:** James Moore, Ivan Osinnii, Amandine Grimm, Björn Oettinghaus, Anne Eckert, Stephan Frank, Erik C. Böttger

**Affiliations:** 1 Institut für Medizinische Mikrobiologie, Universität Zürich, 8006 Zürich, Switzerland; iosinnii@imm.uzh.ch; 2 Transfaculty Research Platform Molecular and Cognitive Neuroscience, Universität Basel, 4055 Basel, Switzerland; amandine.grimm@upk.ch (A.G.); anne.eckert@upk.ch (A.E.); 3 Institut für Pathologie, Universitätsspital Basel, 4031 Basel, Switzerland; bjoern.oettinghaus@gmail.ch (B.O.); stephan.frank@usb.ch (S.F.)

**Keywords:** error-prone translation, ribosomal misreading, mistranslation, proteostasis, liver, ER-UPR, ER stress, Sirtuin-1, RNA-Seq

## Abstract

Translational errors frequently arise during protein synthesis, producing misfolded and dysfunctional proteins. Chronic stress resulting from translation errors may be particularly relevant in tissues that must synthesize and secrete large amounts of secretory proteins. Here, we studied the proteostasis networks in the liver of mice that express the *Rps2*-A226Y ribosomal ambiguity (*ram*) mutation to increase the translation error rate across all proteins. We found that *Rps2*-A226Y mice lack activation of the eIF2 kinase/ATF4 pathway, the main component of the integrated stress response (ISR), as well as the IRE1 and ATF6 pathways of the ER unfolded protein response (ER-UPR). Instead, we found downregulation of chronic ER stress responses, as indicated by reduced gene expression for lipogenic pathways and acute phase proteins, possibly via upregulation of Sirtuin-1. In parallel, we observed activation of alternative proteostasis responses, including the proteasome and the formation of stress granules. Together, our results point to a concerted response to error-prone translation to alleviate ER stress in favor of activating alternative proteostasis mechanisms, most likely to avoid cell damage and apoptotic pathways, which would result from persistent activation of the ER and integrated stress responses.

## 1. Introduction

Accurate protein synthesis is central for the efficient translation of genomic information into functional proteins. However, protein translation is inherently error prone, with errors in mRNA decoding by the ribosome resulting in the missense incorporation of near-cognate aa-tRNAs, reportedly occurring at every one per 10^3^–10^4^ codons [1]. At this rate, around one-fifth of average length proteins (~400 codons) will contain a missense substitution, with protein misfolding as a common outcome [2,3,4].

A proteostasis network including chaperones and protein degradation systems, such as the ubiquitin-proteasome system, is known to facilitate folding or remove terminally misfolded and aggregated proteins [5]. In addition, different cellular compartments are equipped with protein quality control machineries and systems for sensing and correcting for the presence of misfolded proteins, such as the unfolded protein response (UPR) in the endoplasmic reticulum (ER). The ER-UPR consists of three major branches: (i) PERK, upstream of eIF2a; (ii) IRE1, upstream of XBP1; and (iii) ATF6 [6]. Together, these co-ordinate transcriptional and translational programs in response to protein misfolding that reduce protein synthesis, increase protein folding capacity, and promote ER-associated protein degradation [7]. However, while transient activation of the ER-UPR restores proteostasis, persistent activation initiates apoptotic pathways, as seen in response to several acute pharmacological insults that induce severe protein folding defects in the ER [8]. In addition, the accumulation of misfolded proteins in the ER causes ER stress, which is associated with chronic diseases, such as steatosis, activation of systemic inflammatory pathways, and acute phase responses [9,10,11].

The integrated stress response (ISR) is another more general adaptive response to stress conditions, including the accumulation of misfolded proteins in the ER. The ISR overlaps with the ER-UPR via the PERK pathway and serves to restore proteostasis by regulating the rate of translation initiation [12]. The salient feature of ISR activation is phosphorylation of eIF2a in response to stress [13], resulting in a general reduction in mRNA translation, and increased translation of some specific mRNAs, including ATF4, GADD34, and Bim. In the liver, ISR activation also promotes lipogenic pathways compartmentalized in the ER, including fatty acid elongation, cholesterol biosynthesis, and triglyceride biosynthesis, through the activation of SREBP transcription factors [14,15,16]. Over time, activation of the ISR in liver fosters hepatosteatosis and diminished glucose tolerance [16].

Response pathways in the proteostasis network—particularly the ISR and the ER-UPR—have been studied mostly by targeted gene deletion experiments or by selective activation of specific sensors, e.g., PERK activation along with eIF2a phosphorylation. While these studies have yielded great insights, the narrow and acute manipulation of the proteostasis network does not reflect more widespread and longer-lasting stress conditions. As such, we require a more holistic view of how the proteostasis network manages a chronic low-level build-up of misfolded protein, such as is seen from translation errors. Here, we studied the proteostatic networks in the liver of mice with error-prone translation, focusing on the activation of proteostatic mechanisms and the effect on ER-UPR/ISR. Liver tissue is of particular interest in the study of ER proteostasis because it is rich in ER and synthesizes high levels of secretory proteins [17]. Our previous study of mistranslation in cell culture using HEK 293 cells expressing the *Rps2*-A226Y *ram* mutation revealed that chronic proteostatic stress led to translational slowdown and reduced protein import into the ER, thereby decreasing the ER-UPR and avoiding apoptosis [18]. However, the effects of mistranslation on ER proteostasis in vivo have rarely been studied.

Here, we made use of heterozygous knock-in *Rps2*-A226Y mice (hereafter referred to as A226Y mice), which express the ribosomal ambiguity (*ram*) mutation *Rps2*-A226Y [19]. Functionally, the RPS2 protein forms an interface with RPS9 on the small ribosomal subunit. During domain closure, induced by cognate tRNA binding, this interface is disrupted, and the two proteins move apart to allow a conformational change [1]. As with other *ram* mutations, *Rps2*-A226Y disrupts this interface and reduces the energetic cost of domain closure, increasing the probability that near-cognate tRNA binding could also induce domain closure, thus lowering translational accuracy [1,20,21]. *Rps2*-A226Y increases the frequency of stochastic near-cognate missense amino acid incorporation, resulting in elevated levels of misfolded proteins [18]. We used RNA-Seq to profile the liver transcriptome of A226Y mice, and biochemical analysis to investigate the activation of the ISR/ER-UPR and other proteostasis mechanisms in the liver. We observed an absence of the ISR and a downregulation of the ER stress responses in A226Y mice, and simultaneous reshaping of gene expression to activate alternative proteostasis mechanisms. Our results highlight potential adaptations of the proteostasis system under long-term stress, to circumvent chronic activation of the ER-UPR and ISR in favor of less detrimental pathways, including stress granule formation and proteasome degradation.

## 2. Materials and Methods

### 2.1. Rps2-A226Y Transgenic Mice

The transgenic mouse strains *Rps2^A226Y/WT^* and *Rps2^loxP/WT^* have been described previously [19]. In brief, the generation of *Rps2*-A226Y mice was by knock-in using a targeting vector containing a transcriptional STOP cassette flanked by *lox*P sites inserted in exon 1 of *Rps2* in the 5′ UTR upstream of the ATG start codon, and a mutated exon 3 (A226Y; GCC-TAC). Integration of the target vector by knock-in will result in heterozygous *Rps2^loxP/WT^* mice that are haploinsufficient for *Rps2* due to the transcriptional STOP cassette, which inactivates the targeted *Rps2* gene locus. Upon Cre induction by mating with CRE deleter mice, the *loxP*-STOP-*loxP* cassette will be excised and the mutant gene expressed.

Linearized targeting vectors were transfected into 129/Sv embryonic stem (ES) cells, which were then isolated and genotyped. Recombined ES cell clones were microinjected into C57BL/6 mice blastocysts and gave rise to male chimeras. Breeding with wild-type C57BL/6 mice produced *Rps2^loxP/WT^*. These mice were backcrossed to C57BL/6 mice for 7 generations. The heterozygous *Rps2^loxP/WT^* line was crossed with C57BL/6 CRE deleter mice (CMV-Cre) to produce the heterozygous *Rps2^A226Y/WT^* line. For each line, mice were genotyped by PCR, Southern blot, and sequencing.

Analysis of 345 pups produced from breeding *Rps2^loxP/WT^* with CMV-Cre mice found 28 with Cre-mediated recombination of *Rps2*-A226Y, although standard Mendelian genetics would predict 86 (25%), and in these 28 pups, recombination was never present in all cells, but only mosaic animals with partial excision of the targeted allele were identified. To attempt segregation of the excised *Rps2^A226Y^* mutant allele from the floxed non-excised *Rps2^loxP^* allele, three of the partially excised heterozygous males were mated one generation further with C57BL/6 wild-type females, but fully excised mice bearing only the *Rps2^A226Y^* allele were not detected in the 168 pups analyzed. The presence of both the *Rps2^loxP^* and the *Rps2^A226Y^* alleles in the *Rps2^A226Y/WT^* mice (referred to as A226Y mutants) was confirmed by Southern blot [19]. Despite analysis of 513 mice in total, mice displaying excision of the *loxP*-STOP-*loxP* cassette in all cells were never identified.

Liver was carefully isolated from female animals at 15 months of age, snap frozen, and stored at −80 °C until further analysis.

### 2.2. RNA Extraction, cDNA Library Preparation, and Sequencing

RNA was extracted from the liver using TRIzol reagent (Invitrogen, Waltham, MA, USA, 15596026) according to the manufacturer’s instructions. The quality of the isolated RNA was assessed using a Bioanalyzer 2100 (Agilent, Santa Clara, CA, USA). Only those samples with a 260 nm/280 nm ratio between 1.8 and 2.1 and a 28S/18S ratio from 1.5–2 were further processed; all samples used for RNA sequencing had RIN (RNA Integrity Number) ≥ 7.5.

RNA sequencing (RNA-seq) was performed by GATC (Konstanz, Germany). Library preparation was performed with an optimized protocol using standard Illumina (San Diego, CA, USA) adapter sequences. Sequencing was performed with Illumina technology HiSeq 4000 (read mode 1 × 50 bp).

### 2.3. Dual-Luciferase Mistranslation Reporter Assay

Mistranslation was assessed as described previously [18,22], with some modifications. Briefly, misreading was determined using the pRM hRluc-hFluc H245R vector, where His245 (CAC codon) of the humanized firefly luciferase (hFluc) was replaced by Arg245 (near-cognate CGC codon or non-cognate AGA codon). Read-through was determined using pRM hRluc-hFluc D357X, where Asp357 (GAC codon) of hFluc was replaced by a UGA nonsense-codon. HEK 293 cells were first transfected with a vector containing either wild-type *Rps2* or *Rps2*-A226Y plus a hygromycin resistance cassette, using TurboFect (Thermo Scientific, Waltham, MA, USA, R0531) according to the manufacturer’s instructions, and then grown for 10–14 days in the presence of hygromycin (100 μg/mL). Surviving cells were collected and allowed to grow evenly on a new plate, then transfected with a misreading or read-through reporter construct. After 24 h of incubation, cells were lysed and luminescence measured with the Dual-Luciferase^®^ reporter assay system (Promega, Madison, WI, USA, E1980) according to the manufacturer’s instructions, using the FLx800 luminometer (BioTek Instruments, Winooski, VT, USA). Renilla luciferase (Rluc) activity was used as an internal control and misreading and read-through were calculated from the ratio of Fluc/Rluc activity. Fluorescence ratios were normalized, such that the mean values of non-transfected HEK 293 cells, measured in parallel, were equal to 100.

### 2.4. Transcriptome Analysis

The quality of the reads was assessed using FastQC and potential contaminations were evaluated with FastQ Screen using bowtie2 v.2.1.0 [23] default parameters. Quantification of gene expression was performed using the RSEM package (version 1.2.18) [24] mapping against the Ensembl 75 annotations derived from the mouse genome assembly GRCm37. Genes not present (<10 counts per gene) in at least 50% of the samples from one condition were discarded from further analyses. For mutation-related changes, comparisons were made between 10 *Rps2*-A226Y-expressing mutants and 8 Cre-recombinase-expressing wild-type mice, all females. Differential gene expression analysis between groups was performed using the R/bioconductor package edgeR [25]. To evaluate functional changes, differentially expressed genes (*p* < 0.05) were mapped to known biological ontologies and gene pathways based on the Gene Ontology (GO), WikiPathways, and KEGG projects using the functional annotation tool EnrichR [26]. Pathways or GO terms with Benjamini–Hochberg FDR-adjusted *p*-values < 0.05 were considered statistically significant. Visualization of the gene expression within KEGG pathways was done using the R/bioconductor package pathview (version 1.24.0) [27].

### 2.5. Western Blot

Liver tissue was lysed on ice in RIPA lysis buffer (150 mM NaCl, 1% Triton X-100, 0.5% sodium deoxycholate, 0.1% SDS, 50 mM Tris pH 8.0) with Roche cOmplete protease inhibitor (Sigma-Aldrich, St. Louis, MO, USA, 11697498001) and HALT^TM^ Phosphatase Inhibitor Cocktail (Thermo Scientific, Waltham, MA, USA, 78420). Tissue was disrupted by grinding with a pestle while in the lysis buffer, on ice. Lysates were then centrifuged (15,000× *g*, 10 min), supernatant aspirated, and normalized to the protein concentration as measured using a Micro BCA Protein Assay Kit (Thermo Scientific, Waltham, MA, USA, 23235). Samples were boiled in SDS sample buffer for 5 min and resolved by SDS-PAGE. The specific antibodies used were: from Cell Signaling Technology (CST) (Danvers, MA, USA) IRE1α (#3294), eIF2α (#2103), phospho-eIF2α (Ser51) (#9721), ATF6 (#65880), ATF4 (#11815), SirT1 (#9475), Bim (#2933), Akt (#9272), phospho-Akt (Ser473) (#4060), 4E-BP1 (#9644), phospho-4E-BP1 (Thr37/46) (#9459) and eIF4E (#9742); from Thermo Scientific (Waltham, MA, USA) GADD34 (PA1-139); and from Abcam (Cambridge, UK) phospho-IRE1α (Ser724) (ab48187), GRP78 BiP (ab21685), anti-beta Tubulin (ab6046), Anti-Rabbit IgG (HRP) (ab205715) and Anti-Mouse IgG H&L (HRP) (ab6728). Full Western blot gel images are provided in Supplementary Materials Figures S10–S13. Akt control cell extracts (CST, #9273) and HEK 293 cell lysate (lysed in RIPA lysis buffer with protease and phosphatase inhibitors) were used as controls as indicated in the Supplementary Materials Figures S10, S11 and S13. Densitometry was measured using Image Lab software from Bio-Rad (Hercules, CA, USA), and a two-sided unpaired Student’s *t*-test was used to determine *p*-values.

### 2.6. qRT-PCR

The level of XBP1 splicing was measured by qRT-PCR using XBP1-specific primers that amplify spliced and un-spliced XBP1 mRNA. mRNA levels of Sirt1, C2, and Mup6 were corroborated by qRT-PCR using specific amplification primers. RNA samples were reverse transcribed into cDNA using the High-Capacity RNA-to-cDNA Kit (Applied Biosystems, Waltham, MA, USA, 4387406) according to the manufacturer’s instructions. cDNA was analyzed using a 7500 Fast Real-Time PCR System (Applied Biosystems, Waltham, MA, USA) and a pair of gene-specific primers for each selected gene. qPCR was performed with 5x EvaGreen QPCR Mix (Bio&Sell, Feucht, Germany, BS76.590) and 20ng of cDNA per reaction. The transcript levels of target genes were normalized to the mean transcript levels of housekeeping genes (GAPDH, Rpl41, Actb) from the same sample as an internal control, and the fold change of A226Y mutant relative to WT was calculated as 2^−ΔΔCT^. The following primers were used: un-spliced XBP1 forward 5′-CAG CAC TCA GAC TAT GTG CA-3′; spliced XBP1 forward 5′-AGT CCG AAT CAG GTG CAG -3′; spliced & un-spliced XBP1 reverse 5′-GTC CAT GGG AAG ATG TTC TGG-3′; Sirt1 forward 5′-GGA GCA GAT TAG TAA GCG GCT TG-3′, reverse 5′-GTT ACT GCC ACA GGA ACT AGA GG-3′; C2 forward 5′-CAG GAT GTG ACG GAG GTG ATC A-3′, reverse 5′-AGG CGA TCC ATC TGG CTT TGC A-3′; Mup6 forward 5′-GGA AAC CTT CCA GCT GAT GTC G-3′, reverse 5′-CTC TAA TGA TTC CAT GCT CCT CAC-3′; GAPDH forward 5′-CAT CAC TGC CAC CCA GAA GAC TG-3′, reverse 5′-ATG CCA GTG AGC TTC CCG TTC AG-3′; Rpl41 forward 5′- GCC ATG AGA GCG AAG TGG -3′, reverse 5′- CTC CTG CAG GCG TCG TAG -3′; Actb forward 5′-CCT CCC TGG AGA AGA GCT ATG-3′, and reverse 5′-TTA CGG ATG TCA ACG TCA CAC-3′. An unpaired two-sided Student’s *t*-test was used to determine the *p*-value.

### 2.7. TaqMan RT-PCR

The relative ratios of mouse wild-type Rps2 and Rps2-A226Y mRNA were measured using Taqman RT-PCR, with primers flanking the site of mutation (forward 5′-GGT GAC AGG CCG CTG TGG CTC TGT GCT GGT-3′, reverse 5′-AAG TTG CCC AGG GTG GCA GTG CAG-3′) and TaqMan probes specific for wild-type *Rps2* (5′-TGC TAC ACT TCA GCC-3′, NED) or Rps2-A226Y (5′-CTA CAC TTC ATA CAG AG-3′, FAM). Experiments were conducted using a TaqMan™ kit (Thermo Scientific, Waltham, MA, USA, 4352405) and the 7500 Fast Real-Time PCR System (Applied Biosystems, Waltham, MA, USA); amplification of 40 cycles (95 °C for 20 s and 60 °C for 45 s). The 2^−ΔΔCT^ method was used to calculate the ratio between wild-type and mutant mRNA.

### 2.8. Proteasome Activity Assay

Liver tissue was lysed with a pestle in ice-cold lysis buffer (50 mM Tris–HCl (pH 7.5), 150 mM NaCl, 5 mM EDTA, 0.5% NP40, 2 mM DTT) and rotated at 4 °C for 1 h. After centrifugation (16,000× *g*, 10 min, 4 °C), the protein concentration of the supernatant was determined using the Micro BCA Protein Assay Kit (Thermo Scientific, Waltham, MA, USA, 23235). The chymotrypsin activity assay was performed with 15 μg of total protein in 96-well plates, with 50 μM Suc-Leu-Leu-Val-Tyr-AMC peptide substrate (Adipogen Life Sciences, San Diego, CA, USA, AG-CP3-0016) (200 μL total). Fluorescence (excitation 380 nm, emission 460 nm) was measured after 1 h of incubation at 28 °C using a microplate fluorometer Cytation 5 (BioTek Instruments, Winooski, VT, USA). In parallel, the same test was performed in the presence of 100 µM MG-132 (Sigma-Aldrich, St. Louis, MO, USA, M7449) to inhibit the proteasome, and the resulting fluorescence was taken as the non-proteasome specific degradation activity and subtracted from the first fluorescence values. Mean values were taken from six technical replicates. Due to rare occurrences of erroneously high fluorescence measurements across replicates, values above the inter-quartile range of the replicate mean for each sample were removed. A two-sided unpaired Student’s *t*-test was used to determine the *p*-value.

### 2.9. Stress Granule Assay

Equal amounts of liver tissue were ground using a pestle in ice-cold lysis buffer (50 mM Tris pH 7.6, 50 mM NaCl, 5 mM MgCl_2_, 0.1% NP-40, 1x protease inhibitor cocktail (Roche cOmplete (Sigma-Aldrich, St. Louis, MO, USA, 11697498001))) and rotated at 4 °C for 1 h. Lysate was centrifuged at 2000× *g* for 2 min to remove debris and nuclei. The cytosolic supernatant was collected without disrupting the nuclei pellet. After measuring the protein concentration using the Micro BCA Protein Assay Kit (Thermo Scientific, Waltham, MA, USA, 23235), equal amounts of total protein were centrifuged at 10,000× *g* for 10 min to separate the soluble cytosolic fraction from the insoluble ribonuclueoprotein granule fraction (RG). The RG was reconstituted in 6 M urea buffer (6 M urea, 100 mM Tris pH 8.0, 10 mM magnesium acetate, 2% SDS, 10 µM DTT, 1x protease inhibitor cocktail (Roche cOmplete (Sigma-Aldrich, St. Louis, MO, USA, 11697498001)). Samples were boiled in SDS sample buffer for 5 min and resolved by SDS-PAGE. The primary antibodies used were eIF4E (CST, Danvers, MA, USA, #9742) and anti-beta Tubulin (Abcam, Cambridge, UK, ab6046), with Anti-Rabbit IgG (HRP) (Abcam, Cambridge, UK, ab205715) secondary antibody. Densitometry was measured using Image Lab software from Bio-Rad (Hercules, CA, USA), and a two-sided unpaired Student’s *t*-test was used to determine *p*-values.

### 2.10. Preparation of Isolated Mitochondria from Liver

Mitochondria were isolated from the liver as previously described [28]. In brief, liver was dissected on ice, washed in ice-cold PBS 10 mM EDTA, and homogenized with a glass homogenizer (10–15 strokes, 400 rpm) in 1 mL of ice-cold LMI buffer (210 mM mannitol, 70 mM sucrose, 10 mM HEPES, 1 mM EDTA, 0.45% BSA, 0.5 mM DTT, 5× Roche cOmplete protease inhibitor (Sigma-Aldrich, St. Louis, MO, USA, 11697498001)). Liver homogenates were centrifuged at 1450 g for 7 min at 4 °C to remove nuclei and tissue particles; centrifugation was repeated with the supernatant fraction for 3 min. The resulting supernatant fraction was centrifuged at 10,000× *g* for 5 min at 4 °C to pellet mitochondria. The resulting pellet was re-suspended in 1 mL of LMI and centrifuged at 1450× *g* for 3 min at 4 °C to remove debris. The mitochondria-enriched supernatant was centrifuged at 10,000× *g* for 5 min at 4 °C to obtain the mitochondrial fraction. This fraction was re-suspended in 300 μL of PBS, followed by determination of the protein content.

### 2.11. Oxygen Consumption and ATP Measurements in Isolated Mitochondria

Rates of oxygen consumption were measured in isolated mitochondria using a Seahorse Bioscience XF24 Analyzer (Agilent, Santa Clara, CA, USA), following the manufacturer’s protocol and as previously described [29]. Briefly, mitochondria were diluted 1:10 in cold 1 x MAS containing 10 mM succinate, 2 mM malate, and 10 mM pyruvate. In total, 50 μL of mitochondrial suspension (5 μg mitochondrial protein/well) were delivered to each well of a XF Cell Culture microplate (Agilent, Santa Clara, CA, USA) and centrifuged at 2000× *g* for 20 min at 4 °C to let mitochondria adhere to the wells. After centrifugation, 450 μL of pre-warmed (37 °C) 1 x MAS plus substrates were added to each well and the plate was incubated for 5 min at 37 °C in a CO2-free incubator prior to the experiment. The plate was placed in a XF24 Analyzer and oxygen consumption rates were assessed under different respiratory states as described [29], except that oligomycin was added to a final concentration of 2.5 μg/mL.

The ATP content was determined using the Vialight plus kit (Lonza, Basel, Switzerland, LT07-121) following the manufacturer’s instructions and normalization per protein content.

### 2.12. Determination of Superoxide Anion Radicals

MitoSOX™ Red (Thermo Scientific, Waltham, MA, USA, M36008) reagent is a fluorogenic dye that specifically targets mitochondria in live cells. Oxidation of MitoSOX™ Red reagent by superoxide produces red fluorescence. Liver homogenate samples were adjusted to 1 mg protein/mL in HBSS. In total, 150 μL of a 5 μM MitoSOX™ reagent working solution (prepared according to the manufacturer’s protocol) were added to 250 μL of sample, followed by incubation at 37 °C for 10 min, protected from light. Then, samples were centrifuged for 3 min at 500× *g*. After discarding the supernatant, the pellets were washed three times with 250 μL of HBSS (3 min at 500 g). Finally, the samples were transferred into a 96-well plate (final volume of 100 μL per well) and fluorescence was detected using the Victor X5 multiplate reader (PerkinElmer, Waltham, MA, USA) at 510 (excitation) and 580 nm (emission). The intensity of fluorescence is proportional to superoxide anion radicals in mitochondria.

### 2.13. Histopathological Analyses

Liver tissue of *Rps2*-A226Y mutant mice (at 15 months of age) was histologically assessed in comparison to age-matched wild-type littermates (A226Y mutant *N* = 3; WT *N* = 3) (all analyzed animals were females). Following formalin-fixation and paraffin-embedding, 8-μm-thick sections were subjected to the following standard histochemical stains to screen for pathological liver tissue changes [30]: hematoxylin-eosin (H&E) to assess histological abnormalities, such as inflammation or necrosis; D-PAS to detect bile duct integrity; chromotrope aniline blue (*CAB*) and Orcein to assess connective tissue collagen; and Prussian blue to detect iron deposits.

### 2.14. Transcriptome Data Deposition

Transcriptome data are available through the Gene Expression Omnibus (GEO), accession number GSE173101.

## 3. Results

### 3.1. The Rps2-A226Y Mouse Model

The ability of the *Rps2*-A226Y mutation to induce mistranslation, i.e., misreading and stop-codon read-through, as established in [18], was confirmed using a dual-luciferase mistranslation reporter system [18,22] in HEK 293 cells transfected with either *Rps2* wild-type (WT) or *Rps2*-A226Y. To measure misreading, the active site (H245) of firefly luciferase (Fluc) was mutated from CAC to CGC, resulting in a non-functional protein. For read-through, stop-codon TGA was introduced at D357 of Fluc to induce truncation and inactivation. Compared to *Rps2* WT, the synthesis of functional Fluc was significantly higher for *Rps2*-A226Y, for both misreading and read-through, indicating increased mistranslation as a result of the A226Y mutation (Supplementary Materials Figure S1).

The A226Y mouse model is based on a conditional *Rps2*-A226Y allele, which consists of a transcriptional stop-cassette flanked by *loxP* sites inserted into exon 1, and a mutated exon 3 (A226Y). Knock-in integration of the *loxP* allele produces heterozygous *Rps2^loxP/WT^* mice, which are haploinsufficient for *Rps2* due to inactivation of the targeted gene locus by the transcriptional stop-cassette (see Supplementary Materials Figure S2c). To obtain *Rps2^A226Y/WT^* mice, heterozygous *Rps2^loxP/WT^* mice were crossed with a CMV-Cre transgenic mouse line expressing Cre recombinase ubiquitously under control of the CMV promoter, resulting in heterozygous *Rps2^A226Y/WT^* mice. However, Cre-mediated excision in the *Rps2^A226Y/WT^* mice was never complete, such that recombination of the *Rps2* gene locus was present in all cells, as only mice with partial excision of the targeted allele were identified. This resulted in genetic mosaicism of the targeted *Rps2* gene locus in heterozygous *Rps2^A226Y/WT^* mice, with the presence of both non-excised floxed (*Rps2^loxP/WT^*) cells and fully excised A226Y mutant (*Rps2^A226Y/WT^*) alleles [19]. Across the liver of A226Y mutant mice, levels of mutant A226Y mRNA corresponded to approximately 25% of total *Rps2* mRNA, on average (Supplementary Materials Figure S2d).

RNA-Seq was performed on liver tissue and comparisons were made between A226Y mice and Cre-recombinase expressing wild-type control mice (WT). Global transcriptome analysis revealed that with a *p*-value < 0.05, 3897 genes were differentially regulated in the A226Y mutants compared to wild type (Supplementary Materials Figure S2). To control for a possible effect of *Rps2* haploinsufficiency in A226Y mutants as a result of mosaic expression of *Rps2*-A226Y in the liver, we compared gene expression in haploinsufficient *Rps2^loxP/WT^* mice with the appropriate non-haploinsufficient wild-type controls. Compared to A226Y mice, *Rps2^loxP/WT^* mice showed low numbers of differentially expressed genes relative to wild type (FDR-adjusted *p*-value < 0.05 found 11 in *Rps2^loxP/WT^* compared to 1890 in A226Y) and very little overlap in those regulated genes (Supplementary Materials Figure S3). This clear difference indicates independent and pronounced effects of the A226Y mutation beyond any possible haploinsufficiency effect.

### 3.2. Proteostatic Response—Absence of ER-UPR and ISR Activation in A226Y Liver

**Figure 1:**
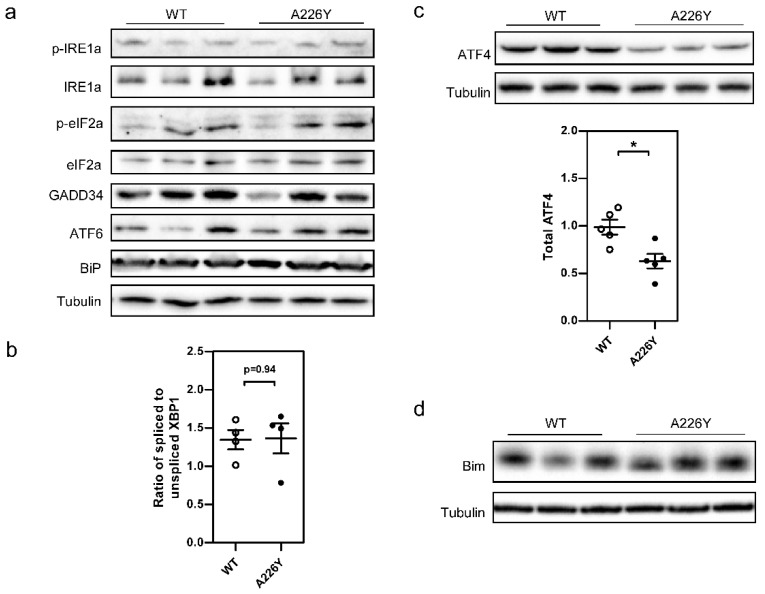
Absence of UPR and ISR activation in the liver of mistranslating A226Y mice. (**a**) Western blots showing total and specific phosphorylation levels for key players in the three branches of the ER-UPR. Significance level for comparison between A226Y and WT (*N* = 5): p-IRE1 (Ser724)/IRE1a *p* = 0.43; p-eIF2a (Ser51)/eIF2a *p* = 0.55; GADD34 *p* = 0.77; ATF6 *p* = 0.48, BiP *p* = 0.64. Densitometry values were normalized to tubulin as the loading control. (**b**) qRT-PCR quantification of spliced XBP1 relative to un-spliced XBP1 (*N* = 4, *p* = 0.94). (**c**) Western blot of total ATF4 protein normalized to tubulin (*N* = 5, *p* = 0.01). (**d**) Western blot of total Bim protein normalized to tubulin (*N* = 5, *p* = 0.45). Plots show mean, SEM, and individual data points. * *p* < 0.05, calculated by unpaired two-sided Student’s *t*-test. *N* = number of independent mice in each comparison. Full gel images are provided in the Supplementary Materials.

Focusing on the transcriptome of A226Y mice, we used enrichment tools to characterize the gene pathways differentially regulated. Reflecting mistranslation-induced disturbances in the ER, we noted enrichment in the term ‘protein processing in endoplasmic reticulum mmu04141′ (adj. *p* = 0.0084). However, we found no increase in terms relating to ER-UPR or ISR. We performed Western blot experiments using specific antibodies to assess total protein and phosphorylation levels of key components of the ER-UPR and found no difference in the activation of the key sensors of misfolded proteins in the ER. IRE1 phosphorylation, eIF2a phosphorylation, and total ATF6 protein levels were comparable in A226Y mutants and WT control animals (Figure 1a). This absence of UPR activation was corroborated by analysis of downstream effectors. Downstream of IRE1, we found no difference in XBP1 splicing as measured by qRT-PCR (Figure 1b) or in total BiP expression (Figure 1a), and downstream of eIF2a, we found unaltered GADD34 expression (Figure 1a) together with markedly decreased expression of ATF4 (Figure 1c).

We measured the expression of Bim in A226Y liver to assess the regulation of apoptosis as the potential culmination of UPR activity [31] and found no difference between A226Y and WT mice (Figure 1d).

We also measured activity of the Akt/mTOR pathway, which is decreased under ER-stress conditions to increase autophagy and apoptosis [32]. We found no difference in Akt or 4E-BP1 phosphorylation as a target of mTOR activity (Supplementary Materials Figure S4).

In the absence of an activated UPR/ISR, the transcriptome points to activation of alternative proteostatic responses in A226Y mutants. Upregulation of various mRNA processing terms (‘mRNA surveillance pathway’, ‘regulation of translation’) and pathways relating to mRNA sequestration and translation control (‘cytoplasmic stress granules’, ‘nuclear body’, ‘ribonucleoprotein granule’) suggests the activation of a stress response able to regulate translation and sequester misfolded protein in the cytoplasm. In addition, we see increased expression of the proteostasis mechanisms ‘proteasome’, ‘ubiquitin-dependent ERAD pathway’, and various Golgi components (Table 1).

**Figure 2:**
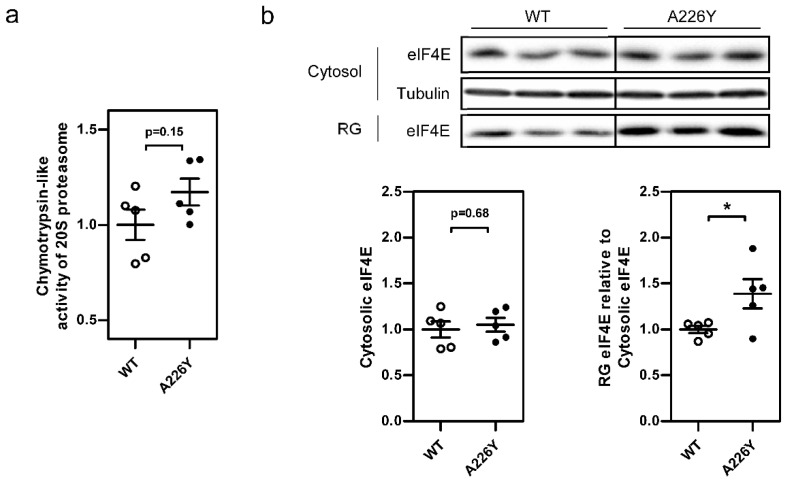
Proteasome activity and stress granule formation in the liver of mistranslating A226Y mice. (**a**) Chymotrypsin-like activity of the 20S proteasome in WT and A226Y liver (*N* = 5). (**b**) eIF4E protein levels in the soluble cytosol fraction and insoluble ribonucleoprotein granule (RG) fraction of WT and A226Y mice liver, as a marker of stress granule formation. Cytosolic eIF4E densitometry values were normalized to tubulin as the loading control (*N* = 5). Plots show mean, SEM, and individual data points. Values in the plots were normalized, such that mean of WT is equal to 1. * *p* < 0.05, calculated by unpaired two-sided Student’s *t*-test. *N* = number of independent mice in each comparison. Full gel images are provided in the Supplementary Materials.

We attempted to clarify the activation of proteostasis mechanisms by measuring 20S proteasome activity and the formation of stress granules in A226Y and WT liver. Chymotrypsin-like proteasomal activity was slightly increased in A226Y mutants, although not statistically significant (*p* = 0.15) (Figure 2a). Stress granule (SG) formation was assessed based on the amount of SG marker protein eIF4E found in the insoluble ribonucleoprotein granule (RG) fraction of liver tissue lysate, relative to soluble cytosolic eIF4E [33]. The increased amount of eIF4E in the RG of A226Y liver indicates increased SG formation (*p* = 0.046) (Figure 2b).

Taken together, these findings testify to the absence of UPR/ISR and the activation of alternative proteostatic responses in mistranslating A226Y liver.

### 3.3. Increased Expression of SIRT1 and Downregulation of ER Stress Responses in A226Y Liver

**Figure 3:**
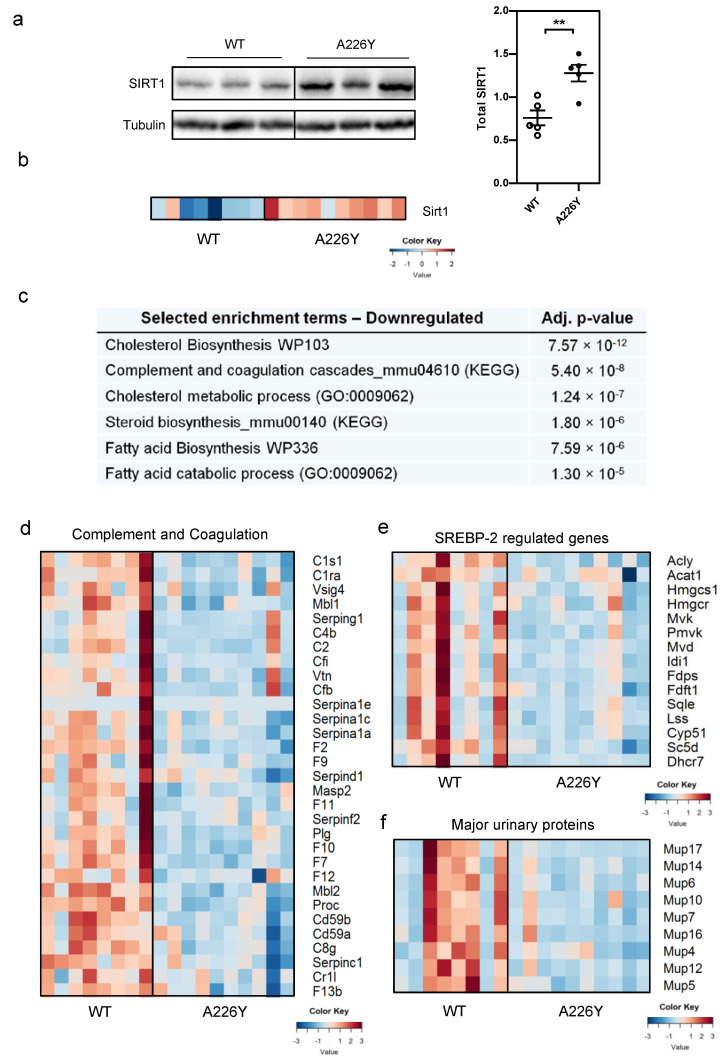
Upregulation of SIRT1 and transcriptional downregulation of the ER stress response in A226Y liver. (**a**) Western blot of total SIRT1 protein normalized to tubulin (*N* = 5, *p* = 0.004). Graph shows mean, SEM, and individual data points. (**b**) Heatmap showing SIRT1 mRNA expression in the liver of WT and A226Y mice (FDR-adjusted *p* = 0.0096). (**c**) Curated gene enrichment list of terms downregulated in A226Y liver relating to the typical ER stress response in liver tissue; FDR-adjusted *p*-values are shown. (**d**) Heatmap showing mRNA expression of downregulated (*p* < 0.05) genes in the KEGG pathway Complement and coagulation cascades_mmu04610. (**e**) Heatmap showing the mRNA expression of genes regulated by SREBP-2, excluding mitochondrial gene variants (gene list taken from [14]). (**f**) Heatmap showing the mRNA expression of regulated major urinary protein (MUP) genes. For transcriptome analysis, *N* = 8 (WT) and *N* = 10 (A226Y). ** *p* < 0.01, calculated by unpaired two-sided Student’s *t*-test. *N* = number of independent mice in each comparison. Full gel images are provided in the Supplementary Materials.

The lifespan-regulating protein Sirtuin-1 (SIRT1) promotes cell survival and is activated in response to cellular stress, including ER stress [34,35]. Several reports have shown that SIRT1 acts as a negative regulator of ER stress and ER stress responses [36,37,38]. Together, this prompted us to study the regulation of SIRT1 in A226Y liver. By RNA-Seq and Western blot, we found significantly increased *Sirt1* gene transcripts and significantly increased SIRT1 protein in A226Y mutants compared to wild-type controls (Figure 3a,b).

In line with the absence of detectable ER-UPR activation and the evident upregulation of SIRT1 expression, we noticed decreased expression of genes involved in the response to ER stress in liver. ER stress increases the acute phase response in the liver and increases steatosis driven by SREBP transcription factors [10,11]. However, we observed downregulation of these processes in the A226Y mutants as illustrated by decreased expression of ‘cholesterol biosynthesis’, ‘steroid biosynthesis’ (Figure 3c), and ‘complement and coagulation cascades’ (Figure 3d, Supplementary Materials Figure S5), combined with reduced SREBP-2 transcriptional activity (Figure 3e). We also found downregulation of gene transcripts for the major urinary proteins (MUPs) (Figure 3f), a group of highly expressed proteins produced by the liver, which act as carriers of pheromones and as metabolic signals to regulate glucose and lipid metabolism, as well as mitochondrial biogenesis [39]. As a control for the RNA-Seq data, we measured the mRNA expression of some relevant genes from these pathways using qRT-PCR, which confirmed the same pattern of expression (Supplementary Materials Figure S6). Collectively, these findings point to increased expression of the lifespan regulator SIRT1 in A226Y liver, together with downregulation of ER stress responses.

### 3.4. Mitochondrial Function in A226Y Liver

**Figure 4:**
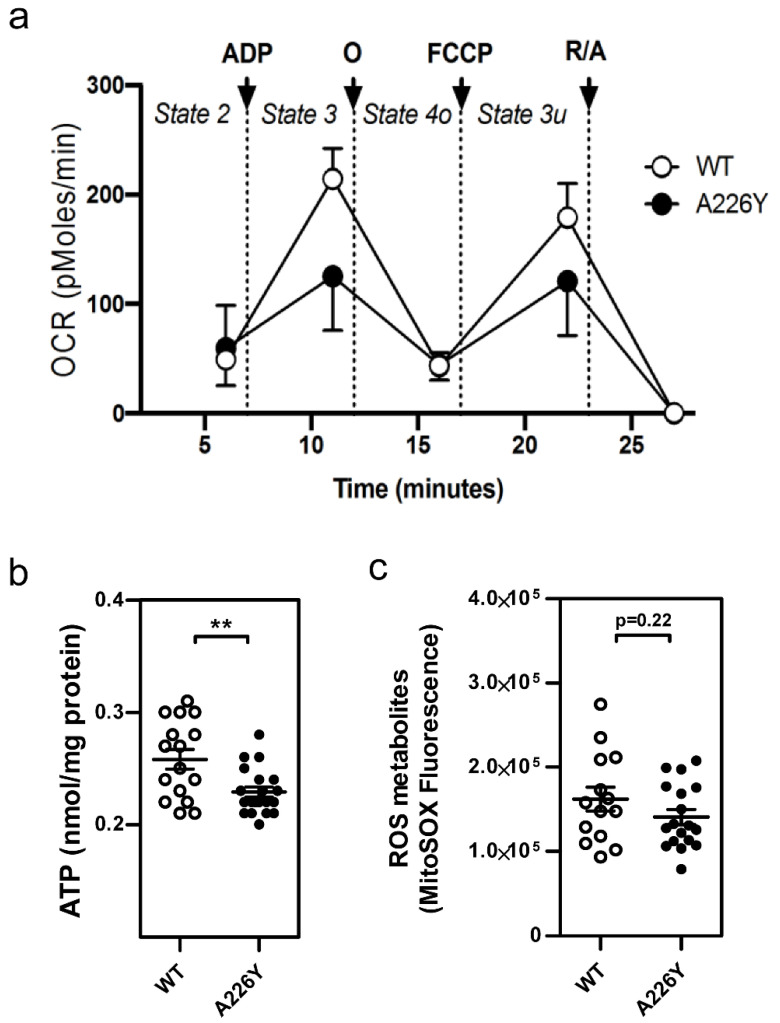
Mitochondrial function in A226Y mouse liver. (**a**) Oxygen consumption rate (OCR) measured under different respiratory states induced by the sequential injection of ADP (to induce state 3), oligomycin (O, to induce state 4o), FCCP (to induce state 3 uncoupled), and rotenone/antimycin A (R/A, to shut down mitochondrial respiration). Graph shows the mean and SD. 8 ≤ *N* ≤ 10, 2 replicates per animal. Two-way ANOVA, multiple comparison between means of WT and A226Y over time; *p* < 0.00001. (**b**) ATP content. Graph shows mean, SEM, and individual data points. 8 ≤ *N* ≤ 10, 2 replicates per animal. (**c**) Mitochondrial superoxide anion radical levels assessed using the fluorescent dye MitoSOX (Ex: 535 nm—Emission: 595 nm). Graph shows mean, SEM, and individual data points. 8 ≤ *N* ≤ 10, 2 replicates per animal. ** *p* < 0.01, calculated by unpaired two-sided Student’s *t*-test. *N* = number of independent mice in each comparison.

We evaluated the capacity of the oxidative phosphorylation system in liver mitochondria of A226Y mice and WT controls using the Seahorse XF24 flux analyzer system (Figure 4a). Total oxygen consumption (OCR) was significantly reduced in the mitochondria of A226Y mice. While basal respiration was unaffected, we observed impaired maximal respiration (state 3, which measures the capacity of mitochondria to metabolize oxygen in the presence of the ATP synthase substrate ADP) and reduced respiration in the absence of a proton gradient (uncoupled state 3u). As a further readout of mitochondrial function, we measured the levels of ATP and ROS (Figure 4b,c). Liver mitochondria from A226Y mutants showed ROS levels comparable to the WT control animals but slightly decreased amounts of ATP. Together, these findings indicate impaired mitochondrial function in A226Y mistranslating liver.

### 3.5. Liver Histopathology

Finally, we performed histopathology analyses to assess possible alterations imposed by the A226Y mutation. Comprehensive histopathological workup using standard histochemical stains to evaluate liver tissue [30] did not show any abnormal changes as compared to wild-type control animals. In particular, we did not find any evidence of increased connective tissue (reflecting potential fibrosis or cirrhosis), nor bile duct abnormalities or pathological intrahepatocyte inclusions (DPAS), or other pathological changes, such as inflammation or hepatocyte necrosis (Supplementary Materials Figure S7).

## 4. Discussion

Under conditions of acute protein misfolding, the ER-UPR and ISR are activated to prevent the accumulation of misfolded protein [6,8,13]. The inability to remove misfolded protein and persistent activation of these responses, however, eventually triggers apoptotic pathways [7,8]. By focusing on the long-term response to an increased production of misfolded proteins in the liver stemming from error-prone translation, we observed how a secretory organ, which must continually produce high quantities of protein, could maintain proteostasis without triggering these apoptotic pathways. To our surprise, we found that the ISR is not activated and that ER stress responses are downregulated, as illustrated by reduced levels of ATF4 and reduced expression of genes associated with ER stress, including acute phase responses and lipid biosynthesis. The downregulation of these pathways highlights how the stress of chronic build-up of misfolded proteins can be alleviated through a concerted response in the liver, which we suggest works through the combined action of upregulation of alternative stress responses together with induction of the longevity regulator SIRT1.

Sirtuin-1 (SIRT1) belongs to a family of nicotinamide adenine nucleotide (NAD^+^)-dependent deacetylases, which exert powerful regulatory effects by means of their ability to interact with or deacetylate a wide range of signaling molecules, transcription factors, and histone proteins [40]. SIRT1 promotes cell survival and is activated in response to cellular stress [40]. SIRT1 has been shown to attenuate ER stress and to downregulate ER stress-induced responses by reducing ER stress-mediated ATF4 and lipid/cholesterol regulator SREBP expression [34,36,38,40]. Reflecting this relationship, we found a significant change in the ATF4/SIRT1 ratio comparing A226Y and wild-type liver (*p* = 0.005) (Supplementary Materials Figure S8). We hypothesize that the increased expression of SIRT1 helps to attenuate ER stress and to dampen ER stress-induced responses in mistranslating A226Y mutant liver. Dampening the ER stress response presumably serves to maintain physiological homeostasis not only by avoiding ER stress-induced apoptosis, but also by counteracting the negative consequences of chronic ER stress stemming from SREBP activity and acute phase protein expression, which would otherwise cause hepatic steatosis and inflammatory disease [9,10,11].

The observed upregulation of alternative stress responses in the liver of A226Y mutants includes increased expression of RNP granules (nuclear bodies, PML bodies, and cytoplasmic stress granules), proteasomal degradation, and Golgi transport. RNP granules are activated in cellular stress responses and can sequester mRNA together with RNA-binding proteins and other translation factors [41,42]. Recent data also point to an extensive interaction between misfolded proteins and RNP granules [43,44,45]. Furthermore, the ubiquitin-dependent proteasome is an established proteostatic response to misfolded protein [46] and is directly involved in degrading misfolded ER proteins, which are exported to the cytosol via ERAD [47]. Biochemical quantification of the stress granule marker protein eIF4E in the insoluble RNP granule fraction of the liver suggested that stress granule formation is increased in A226Y liver. Similarly, proteasome activity was found to be increased in A226Y liver, although it did not reach the *p*-value ‘significance’ cut-off of *p* < 0.05. However, the combined evidence of the transcriptome data and the proteasome activity assay suggests that proteasome degradation is increased in A226Y liver.

Evidence for impaired mitochondrial function in A226Y liver comes from reduced maximal respiration and reduced ATP content, together with decreased mitochondrial gene expression. One possible explanation for impaired mitochondrial function in the context of error-prone translation relates to the import of misfolded proteins into mitochondria as a mechanism of proteostasis, which has been shown to occur in response to mistranslation [18,48].

Our findings highlight an adaptive response, which presumably serves to avoid the detrimental consequences of prolonged ISR/ER-UPR activation by their entire downregulation, and instead achieves proteostasis through alternative mechanisms. While further studies on liver function are warranted, the adaptive mechanisms in place can apparently compensate for the translational defects imposed by the A226Y misreading mutation, at least at the level of histopathology, which testifies to the absence of abnormal changes. We hypothesize that the regulation of alternative proteostasis pathways could collectively provide a means to dampen the accumulation of misfolded proteins in the ER, while SIRT1 attenuates ER stress. This allows circumvention of UPR and ISR activation. We suggest that the regulation of nuclear bodies [42] and cytoplasmic stress granules [41,49] could be involved in sequestration of mRNAs to regulate translation, much in the same way as the ISR typically reduces translation via eIF2a activation. In addition to translation regulation, stress granules may also contribute to overall apoptosis avoidance by suppressing stress-responsive apoptotic pathways [50]. Meanwhile, the accumulation of misfolded proteins in the ER is avoided by exporting them via the Golgi [51] or via ERAD for degradation by the ubiquitin-proteasome system [47] (for a summarizing hypothetical scheme, see Supplementary Materials Figure S9).

Interestingly, decreased expression of MUPs and ‘complement and coagulation cascades’ in mice liver was recently described as a prominent genetic signature associated with lifespan extension across various pharmacological, genetic, and dietary interventions [52]. The co-occurrence of this gene expression signature both in longevity and in A226Y mistranslating liver may suggest that these changes represent a pro-survival adaptation to ER stress. Thus, we hypothesize that the interventions that prolong life are in some way associated with a decrease in ER stress in the liver, such as we found in our study.

The observation that ER-UPR can be circumvented despite ongoing mistranslation improves our understanding of the adaptations possible to long-term proteostatic stress in mammals. However, it is not without precedent, as a similar response was recently observed in HEK 293 cells expressing *Rps2*-A226Y [18]. By uncovering a similar adaptation in vivo, we give weight to the idea that long term low-level misfolding protein stress requires proteostasis responses, which avoid prolonged activation of the UPR.

What we found in this study indicates that chronic ER stress can be circumvented to maintain organismal health in the face of continuous and increased synthesis of mistranslated proteins. The gene regulation we observed indicates mechanisms that maintain proteostasis while avoiding the UPR and the detrimental effects of persistent UPR activation. We acknowledge that a more detailed understanding of the underlying mechanisms and signaling pathways is warranted, not the least as a potential therapeutic target for mitigation of ER stress present in chronic liver diseases.

## Figures and Tables

**Table 1:** Gene expression changes indicate the activation of proteostasis mechanisms in A226Y liver. Curated gene enrichment list comparing A226Y and WT liver; FDR-adjusted *p*-values shown.

Selected Enrichment Terms—Upregulated	Adj. *p*-Value
mRNA surveillance pathway_mmu03015 (KEGG)	3.22 × 10^−4^
RISC complex (GO:0016442)	2.95 × 10^−2^
Cytosolic stress granule (GO:0010494)	4.15 × 10^−2^
Ribonucleoprotein granule (GO:0035770)	4.22 × 10^−2^
	
Nuclear body (GO:0016604)	2.41 × 10^−6^
Nucleolous (GO:0005730)	1.22 × 10^−3^
Nuclear speck (GO:0016607)	3.87 × 10^−3^
	
Ubiquitin-dependent protein catabolic process (GO:0006511)	2.02 × 10^−9^
Proteasome_mmu03050 (KEGG)	2.74 × 10^−3^
Ubiquitin-dependent ERAD pathway (GO:0030433)	4.30 × 10^−2^
	
Golgi subcompartment (GO:0098791)	1.66 × 10^−3^
ER to Golgi vesicle-mediated transport (GO:0006888)	8.42 × 10^−3^
**Selected enrichment terms—Downregulated**	**Adj. *p*-value**
Mitochondrion (GO:0005739)	2.13 × 10^−12^
Mitochondrial matrix (GO:0005759)	2.00 × 10^−9^

## Data Availability

RNA-Seq data is available through the Gene Expression Omnibus (GEO) database, accession number GSE173101.

## Supplementary Materials

The following are available online at https://www.mdpi.com/article/10.3390/cells10112856/s1, Figure S1: Protein mistranslation induced by Rps2-A226Y in HEK 293 cells. Figure S2: Transcriptome overview of A226Y vs. WT mouse liver. Figure S3: Comparison of the number of genes regulated in liver of A226Y mice and haploinsufficient *Rps2^loxP/WT^* mice. Figure S4: Western blots showing absence of activation of key components of the Akt/mTOR pathway in A226Y mice liver. Figure S5: Decreased gene expression of the ‘Complement and coagulation cascades’ pathway in A226Y mice liver. Figure S6: qRT-PCR validation of gene expression in A226Y and WT mouse liver. Figure S7: Liver histology of Rps2-A226Y mutant mice at 15 months of age does not reveal any abnormalities. Figure S8: Increased SIRT1 coincides with decreased ATF4 in A226Y mice liver. Figure S9: Hypothetical mechanisms of ER proteostasis in the presence of error-prone translation. Figures S10–S13: Full Western blot gel images.
