# Supplementary material for: Silencing of the ER and Integrative Stress Responses in the Liver of Mice with Error-Prone Translation
Source: Cells. 2021 Oct 23;10(11):2856. doi: 10.3390/cells10112856 (PMC8616113; doi:10.3390/cells10112856)
Supplement: Supplementary file 1 [file cells-10-02856-s001.zip › cells-1395302-supplementary final .pdf]

Supplementary Figure S1

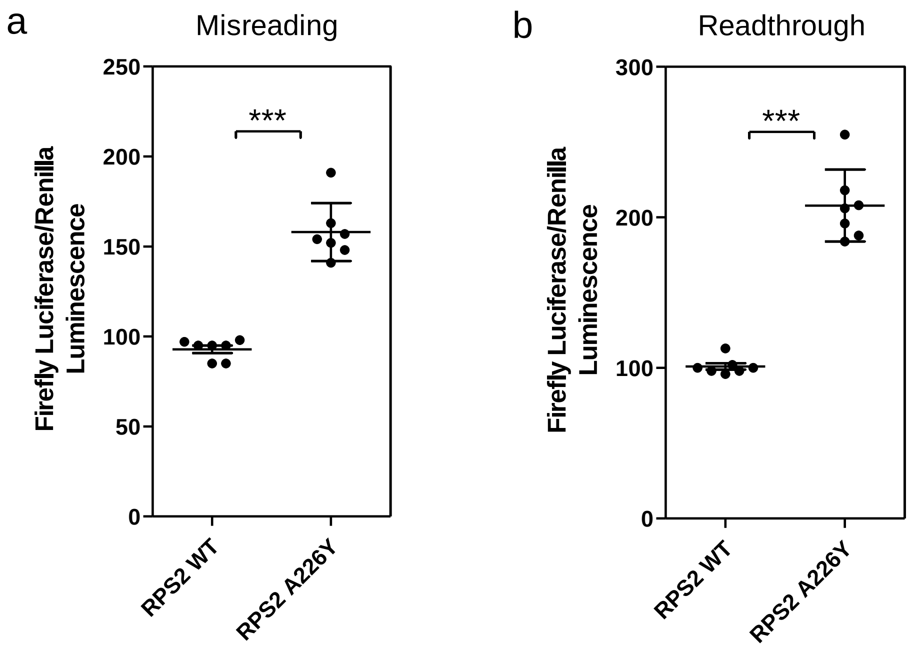

Supplementary Figure S1: Protein mistranslation induced by Rps2-A226Y in HEK 293 cells. (a) Misreading (*N*=7, *p*=7.68x10^{-8}), and (b) STOP-codon readthrough (*N*=7, *p*=3.31x10^{-7}), as measured by Dual Luciferase mistranslation reporter assay. ****p*<0.001. *N* = number of replicate measurements.

Supplementary Figure S2

a

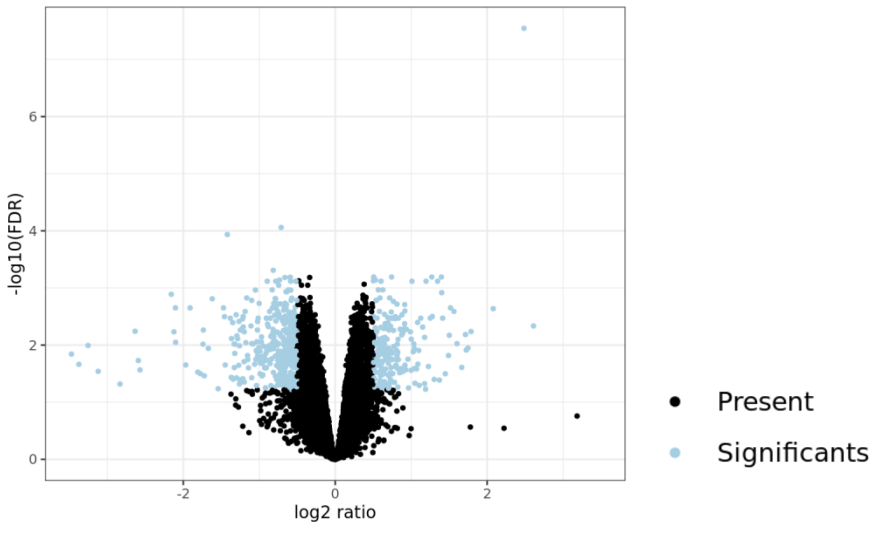

b

p-value	#sig	FDR	fc > 1.5	fc > 2
***p* < 0.05**	3897	0.1699	520	129
***p* < 0.01**	2171	0.0610	441	120
***p* < 0.001**	852	0.0155	268	80

c d

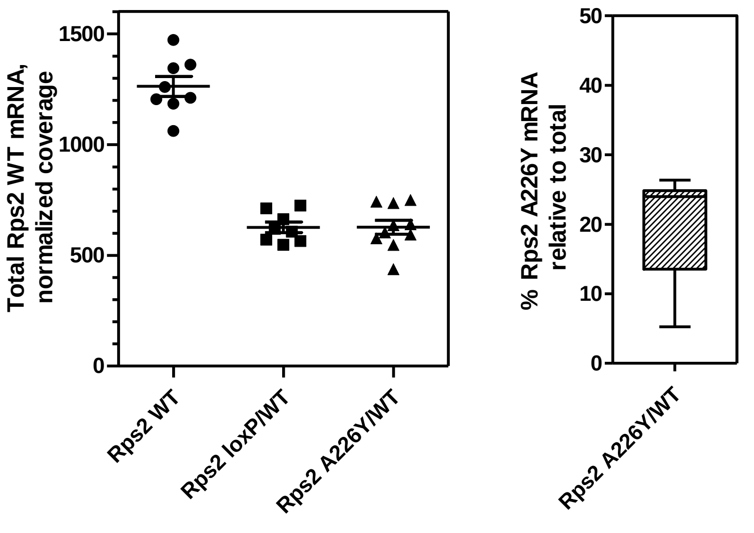

Supplementary Figure S2: Transcriptome overview of A226Y vs WT mouse liver. (a) Volcano plot illustrating the differentially expressed genes. (b) Number of significant differentially expressed genes by *p*-value and fold-change. (c) Plot showing total Rps2 WT mRNA counts in mice liver from Rps2 WT (*N*=8), Rps2 loxP/WT (*N*=8) and Rps2 A226Y/WT (*N*=10) groups, as measured by RNA-Seq. Graph shows mean, SEM and individual datapoints. (d) Expression level of Rps2 A226Y mRNA in Rps2 A226Y/WT mice liver as a percentage of total Rps2 mRNA, as measured by Taqman RT-PCR. Plot shows median, lower and upper quartile, and whiskers 5-95 percentile (*N*=9). *N* = number of independent mice in each comparison.

Supplementary Figure S3

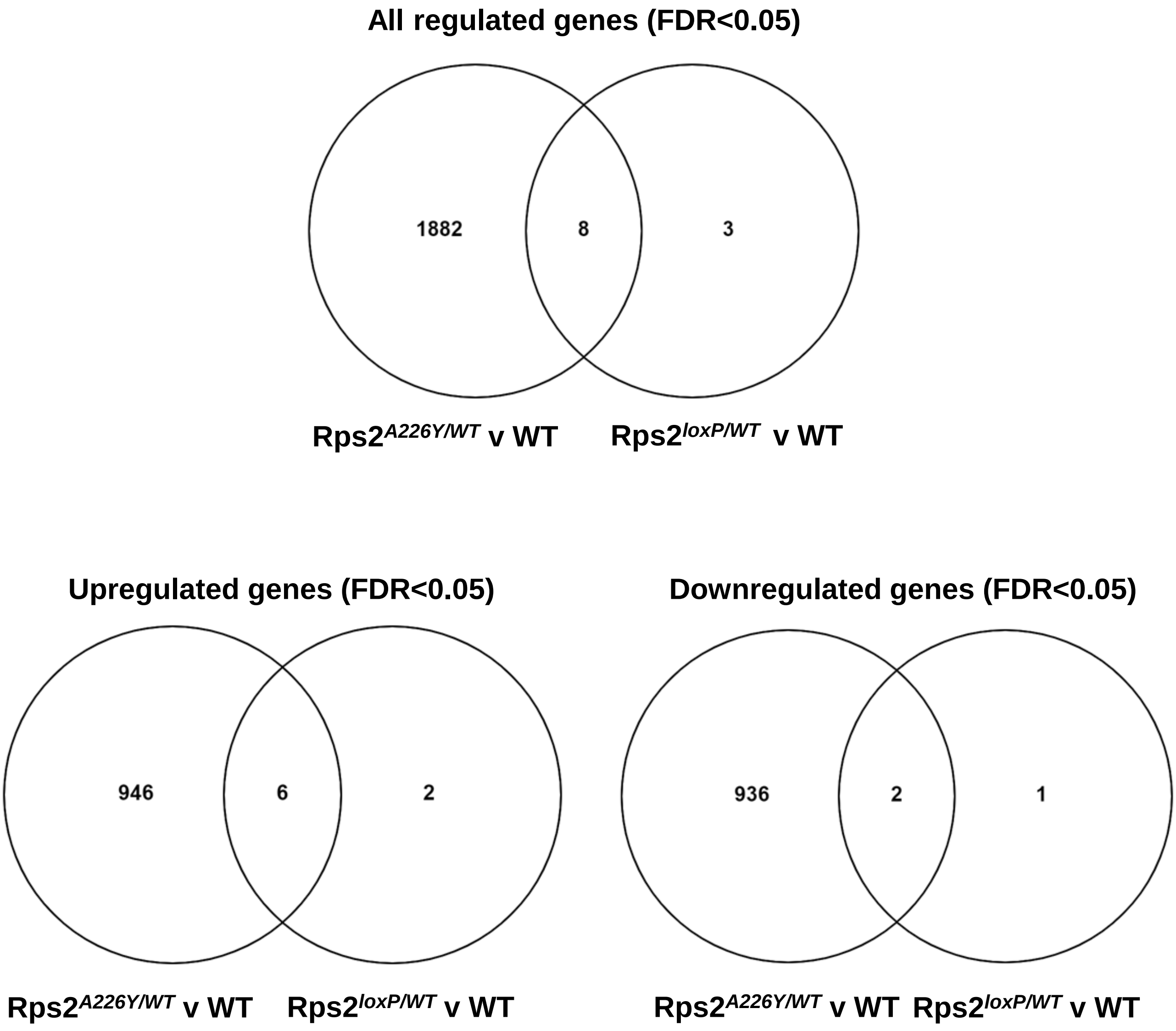

Supplementary Figure S3: Comparison of the number of genes regulated in liver of A226Y mice and haploinsufficient Rps2$^{loxP/WT}$ mice. Comparison between Rps2 A226Y/WT (*N*=10) and Rps2 loxP/WT (*N*=8) groups, relative to WT (*N*=8). Genes were considered significant if they had an FDR adjusted *p*-value < 0.05. *N* = number of independent mice in each comparison.

Supplementary Figure S4

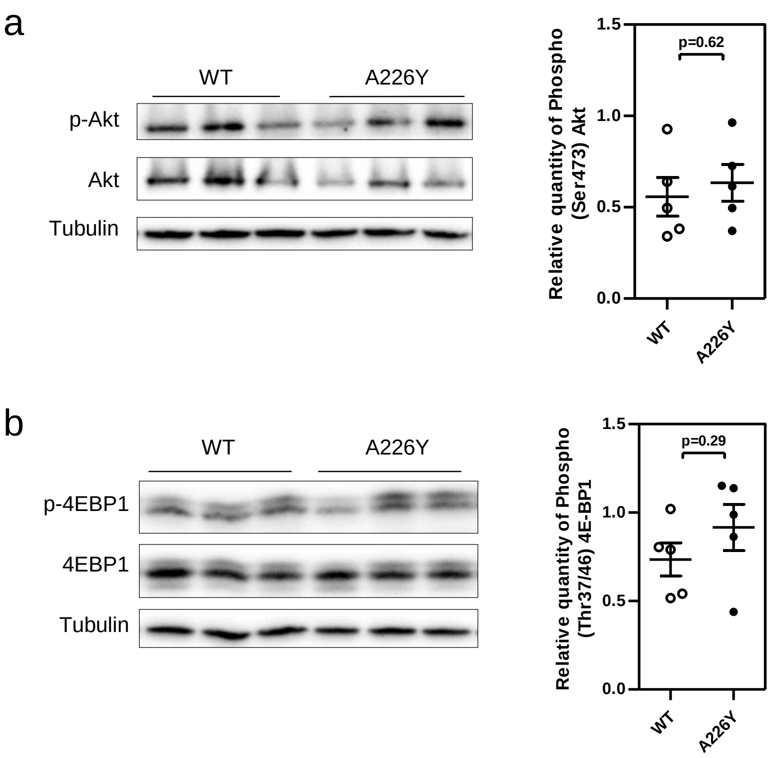

Supplementary Figure S4: Western blots showing absence of activation of key components of the Akt/mTOR pathway in A226Y mice liver. (a) Western blot showing total and specific phosphorylation levels of Akt (*N*=5). p-Akt (Ser473) / Akt comparison between A226Y and WT *p*=0.62. (b) Western blot showing total and specific phosphorylation level of 4EBP1 (*N*=5). p-4EBP (Thr37/46) / 4EBP1 comparison between A226Y and WT *p*=0.29. All densitometry values were normalized to tubulin as loading control. Graphs show mean, SEM and individual data points. *N* = number of independent mice in each comparison.

Supplementary Figure S5

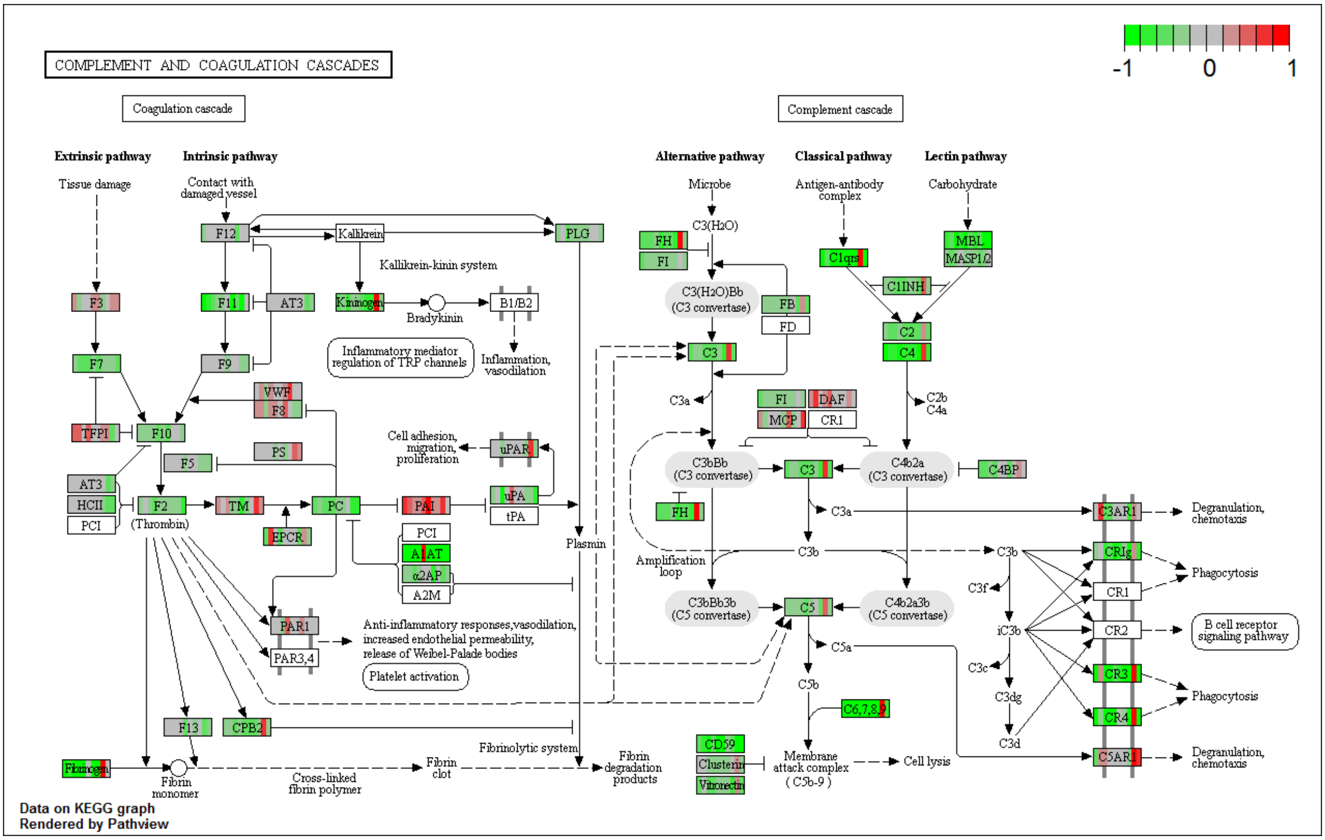

Supplementary Figure S5: Decreased gene expression of the 'Complement and coagulation cascades' pathway in A226Y mice liver. RNA-Seq data visualised upon the KEGG pathway 'mmu04610 Complement and coagulation cascades' using pathview [50], showing the expression of genes in A226Y mice liver (*N*=10) relative to WT mice liver (*N*=8). *N* = number of independent mice in each comparison.

Supplementary Figure S6

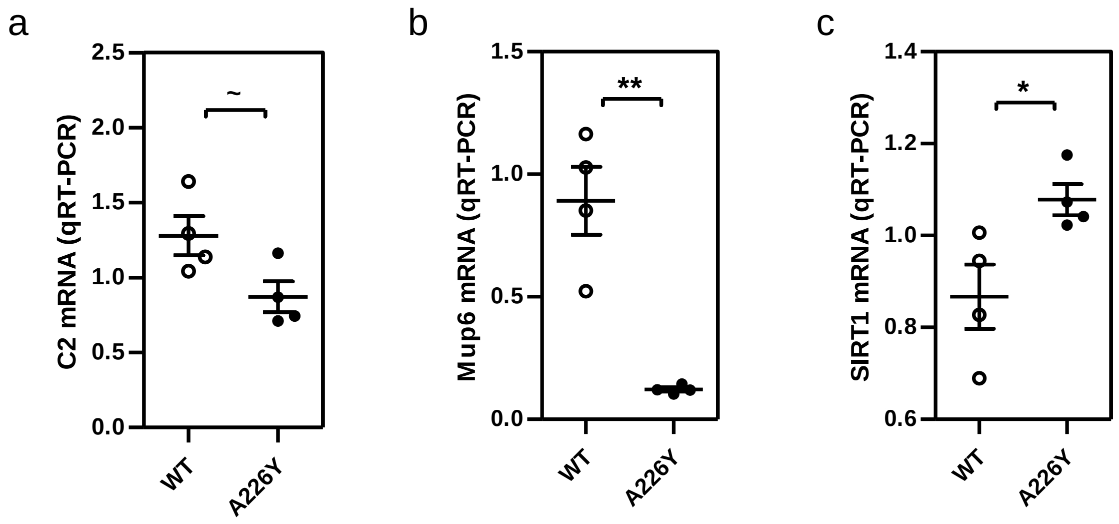

Supplementary Figure S6: qRT-PCR validation of gene expression in A226Y and WT mouse liver.

(a) C2 of the Complement and Coagulation pathway (*N*=4, *p*=0.051). (b) Mup6, an MUP gene (*N*=4, *p*=0.0015). (c) Sirt1 (*N*=4, *p*=0.034). Graphs show mean, SEM and individual data points. ~*p*<0.1, *p<0.05, **p<0.01. *N* = number of independent mice in each comparison.

Supplementary Figure S7

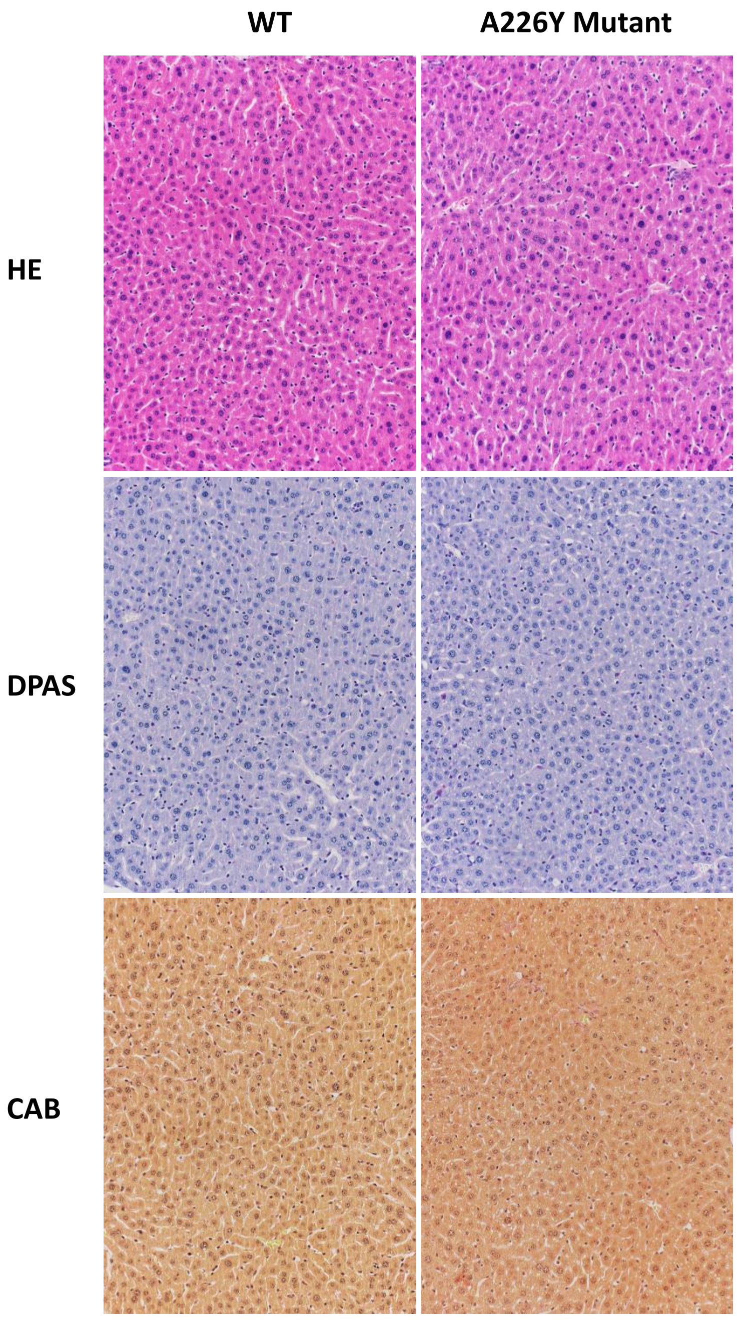

Supplementary Figure S7: Liver histology of Rps2-A226Y mutant mice at 15 months of age does not reveal any abnormalities. No inflammation or hepatocyte necrosis is detectable by H&E staining (top row). No bile duct abnormalities, intrahepatocyte inclusions (DPAS, middle row), or signs of fibrosis or cirrhosis (CAB stains, bottom row) were found.

Supplementary Figure S8

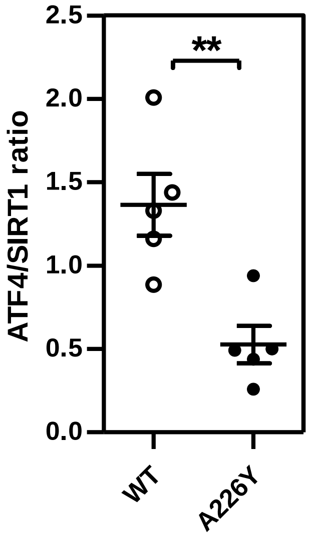

Supplementary Figure S8: Increased SIRT1 coincides with decreased ATF4 in A226Y mice liver. Quantification of protein expression by Western blot densitometry illustrates a shift in the ratio of ATF4 to SIRT1 protein in A226Y mice liver (*N*=5, *p*=0.0048). All values were first normalized to tubulin as loading control. Graph shows mean, SEM and individual data points. ***p*<0.01. *N* = number of independent mice in each comparison.

Supplementary Figure S9

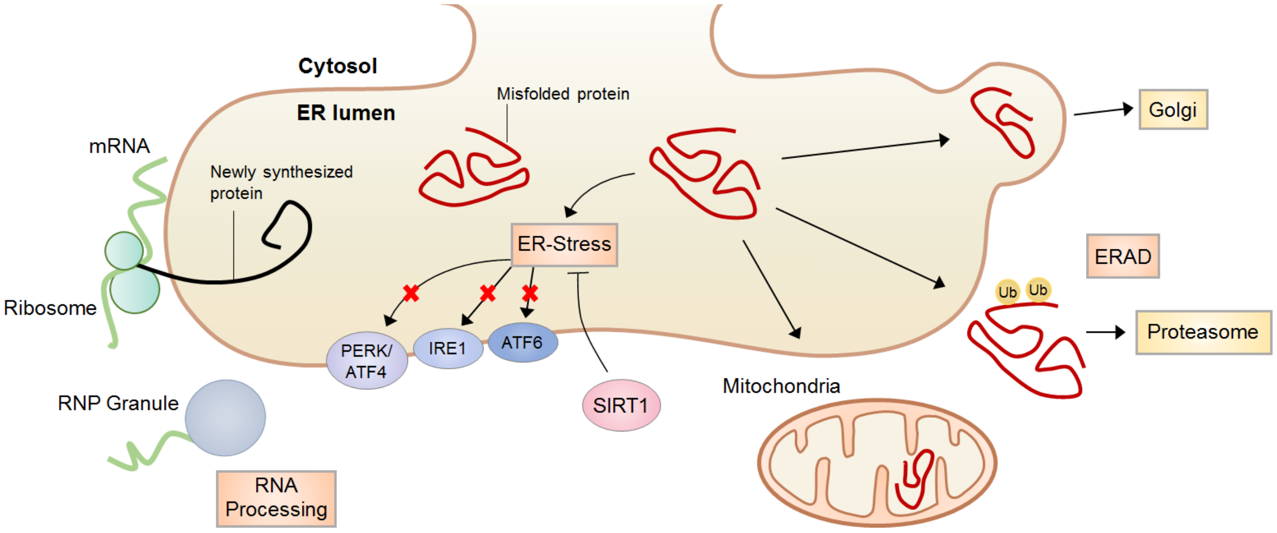

Supplementary Figure S9: Hypothetical mechanisms of ER proteostasis in the presence of error-prone translation. Prolonged activation of the UPR is avoided through the use of alternative proteostatic mechanisms, together with SIRT1 activation to alleviate ER-Stress.

Supplementary Figure S10

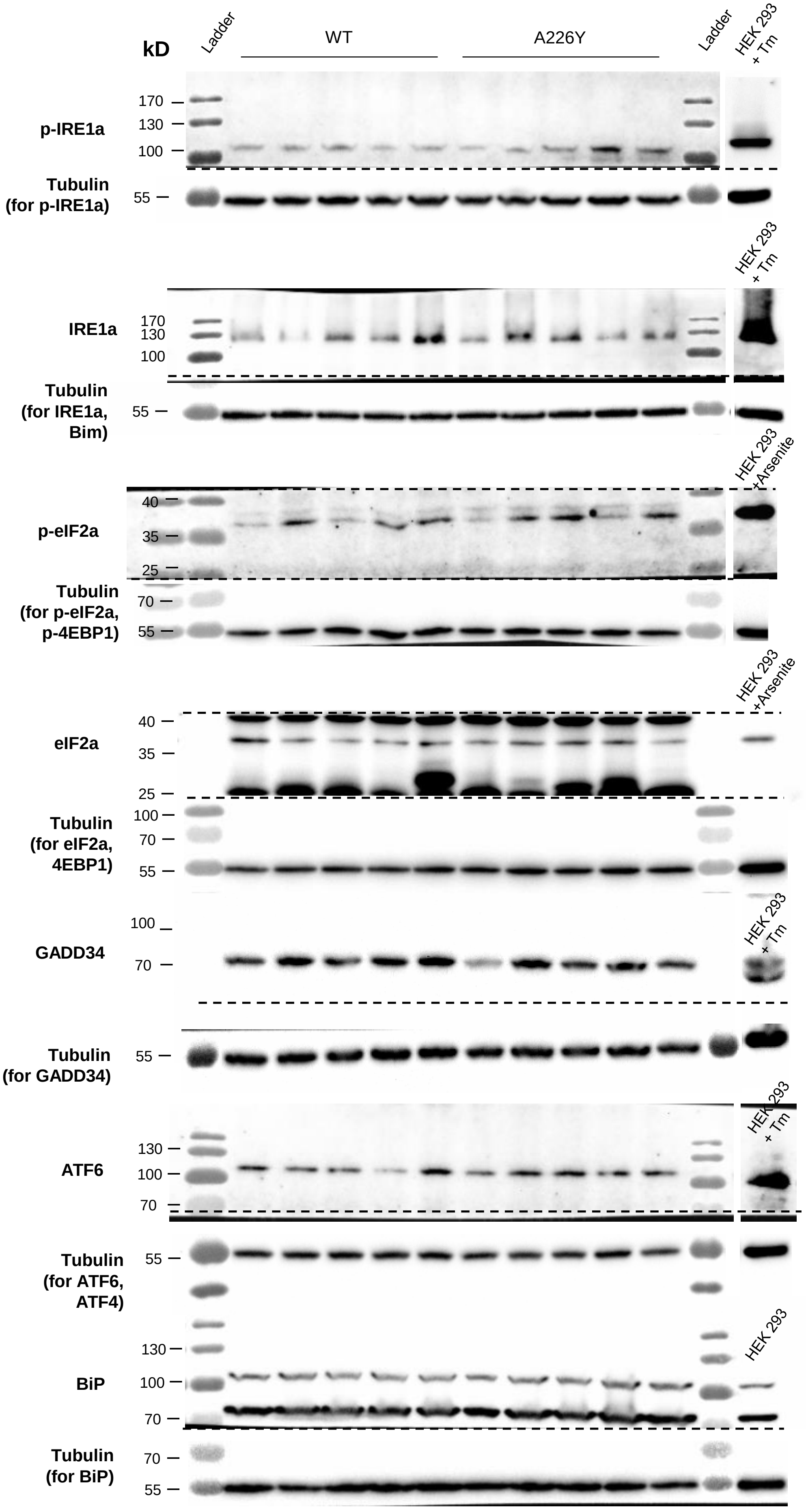

Supplementary Figure S10: Full gel images with corresponding molecular size markers for Figure 1a. Equal amounts of total protein were loaded onto an SDS gel, run and transferred onto a NC membrane. HEK 293 cells, HEK 293 cells treated with tunicamycin (5ug/ml, 16 hours) and HEK 293 cells treated with arsenite (0.5mM, 1 hour) were used as controls where indicated. Tubulin as loading control. eIF2a runs at 38kDa, the additional bands at ~40kDa and 25kDa in the eIF2a blot correspond to endogenous heavy and light chain immunoglobulins (eIF2a was detected with anti-mouse secondary antibody). BiP runs at approximately 78kDa. Tubulin for BiP loaded on a separate gel with same loading and preparation. For p-IRE1a, IRE1a, p-eIF2a, GADD34 and ATF6 images, the corresponding control has been moved closer to test samples for clarity of display. For p-IRE1, a control with a lower exposure is shown. Dashed line marks where the membrane was cut to detect multiple targets simultaneously.

Supplementary Figure S11

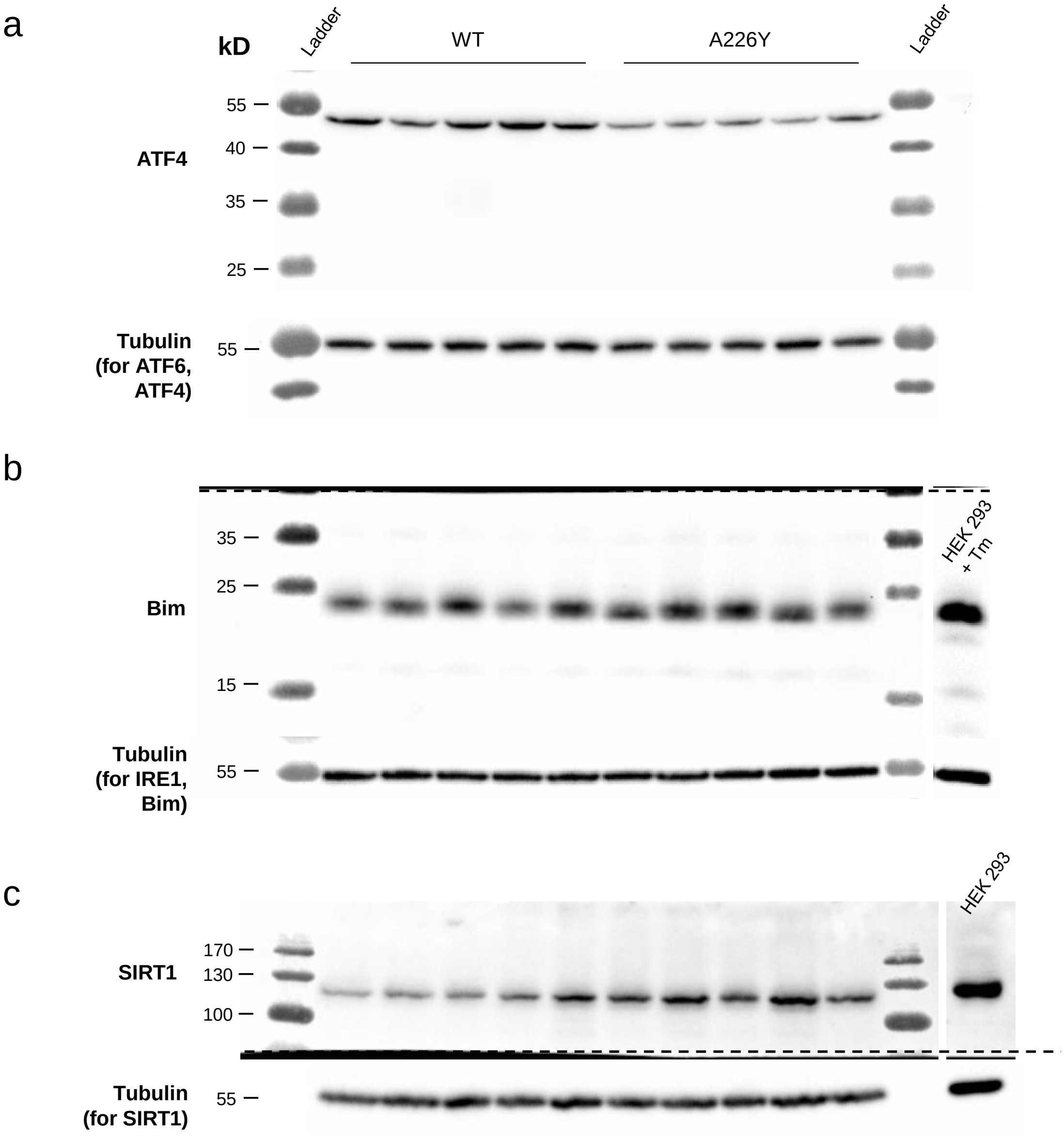

Supplementary Figure S11: Full gel images with corresponding molecular size markers for (a) Figure 1c (b) Figure 1d, and (c) Figure 3a. Equal amounts of total protein were loaded onto an SDS gel, run and transferred onto a NC membrane. HEK 293 cells and HEK 293 cells treated with tunicamycin (5ug/ml, 12 hours) were used as controls where indicated. Tubulin as loading control. Tubulin for ATF4 loaded on a separate gel with same loading and preparation due to molecular size overlap. For Bim and SIRT1 images, the corresponding control has been moved closer to test samples for clarity of display. Dashed line marks where the membrane was cut to detect multiple targets simultaneously.

Supplementary Figure S12

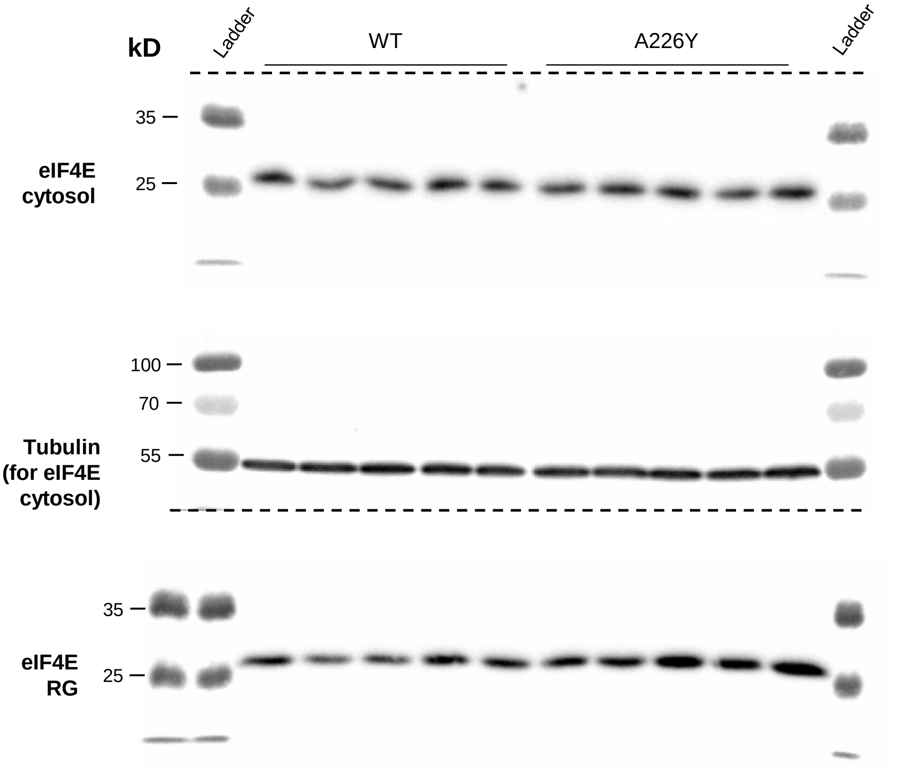

Supplementary Figure S12: Full gel images with corresponding molecular size markers for Figure 2b. Equal volumes of protein lysate were loaded onto an SDS gel, run and transferred onto a NC membrane. Tubulin as loading control for cytosolic eIF4E. Dashed line marks where the membrane was cut to detect multiple targets simultaneously.

Supplementary Figure S13

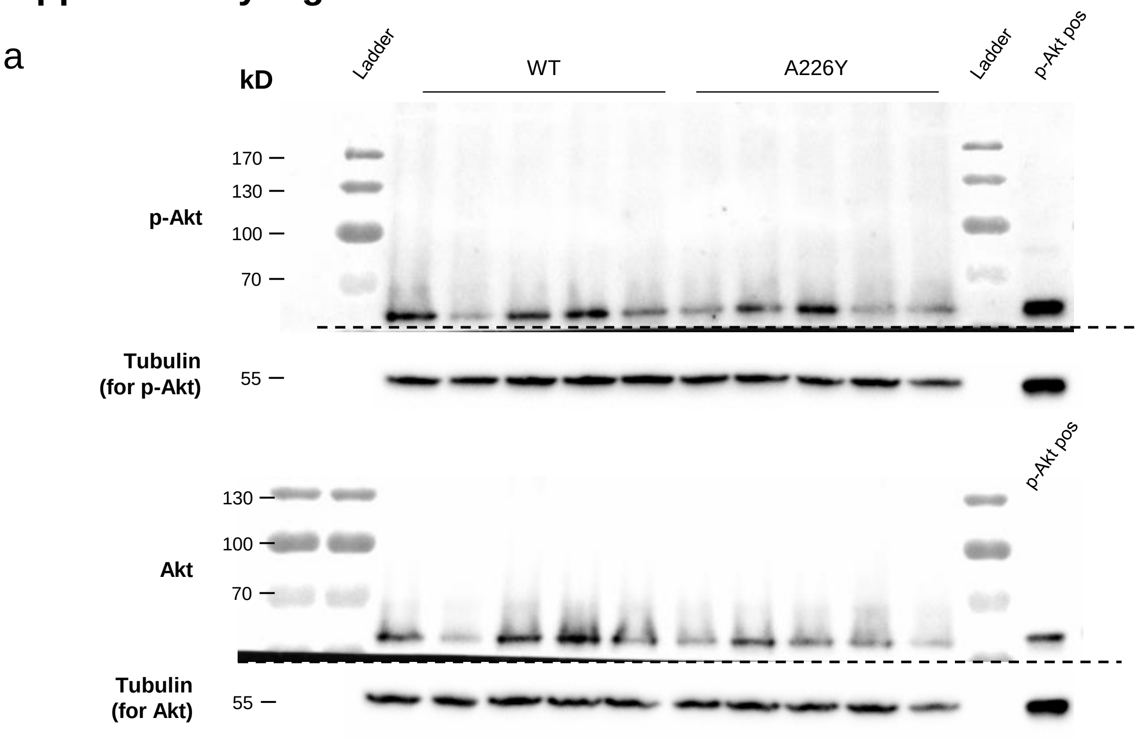

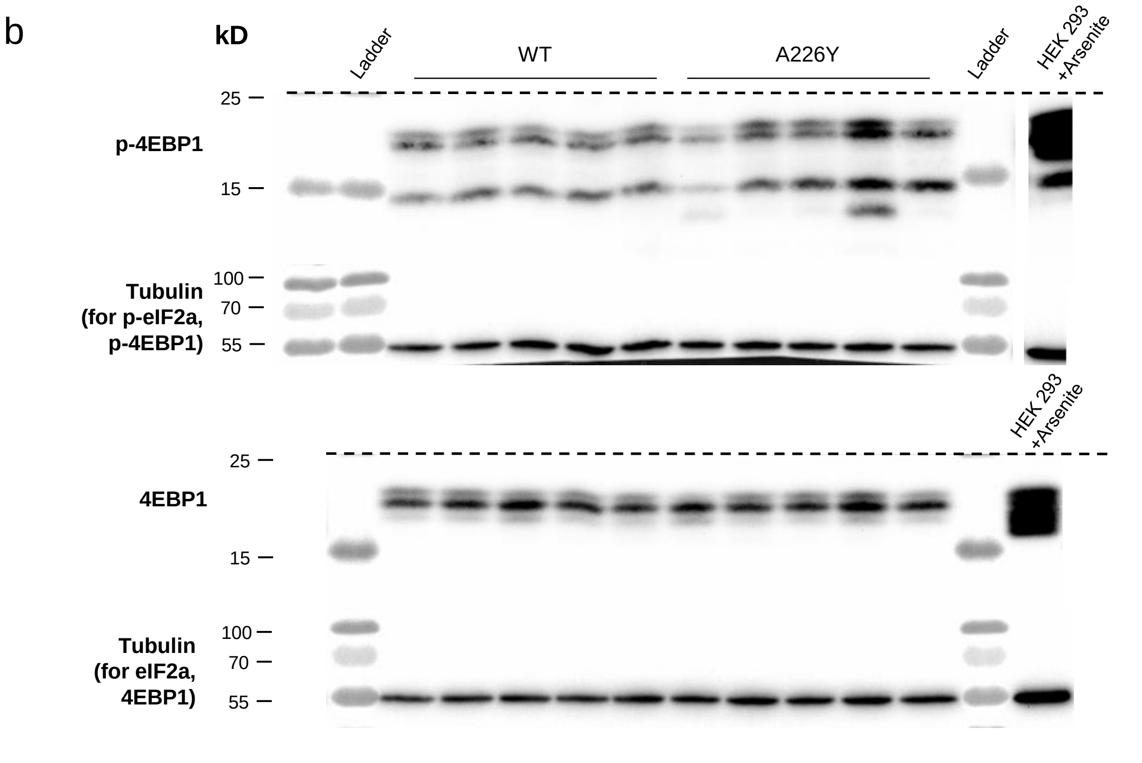

Supplementary Figure S13: Full gel images with corresponding molecular size markers for (a) Supplementary Figure S4a, (b) Supplementary Figure S4b. Equal amounts of total protein were loaded onto an SDS gel, run and transferred onto a NC membrane. HEK 293 cells, HEK 293 cells treated with Arsenite (0.5mM, 1 hour), and Akt control cell extracts from CST (#9273) were used as controls where indicated. Tubulin as loading control. For the p-4EBP1 image, the control has been moved closer to test samples for clarity of display. Dashed line marks where the membrane was cut to detect multiple targets.
